# Choline Kinase: An Unexpected Journey for a Precision Medicine Strategy in Human Diseases

**DOI:** 10.3390/pharmaceutics13060788

**Published:** 2021-05-25

**Authors:** Juan Carlos Lacal, Tahl Zimmerman, Joaquín M. Campos

**Affiliations:** 1 Instituto de Investigaciones Biomédicas, CSIC, 28029 Madrid, Spain; 2 Instituto de Investigación Sanitaria Hospital La Paz, IDIPAZ, 28046 Madrid, Spain; 3 Food Microbiology and Biotechnology Laboratory, Department of Family and Consumer Sciences, College of Agriculture and Environmental Sciences, North Carolina University, 1601 East Market Street, Greensboro, NC 27411, USA; tzimmerman@ncat.edu; 4 Departamento de Química Farmacéutica y Orgánica, Facultad de Farmacia, c/Campus de Cartuja, s/n, Universidad de Granada, 18071 Granada, Spain; 5 Instituto Biosanitario de Granada (ibs. GRANADA), SAS-Universidad de Granada, 18071 Granada, Spain

**Keywords:** phospholipids metabolism, choline kinase, bispyridinium compounds, bisquinolinium compounds, QSAR, anticancer drugs, rheumatoid arthritis, parasites, pathogenic bacteria, inflammatory disease

## Abstract

Choline kinase (ChoK) is a cytosolic enzyme that catalyzes the phosphorylation of choline to form phosphorylcholine (*P*Cho) in the presence of ATP and magnesium. ChoK is required for the synthesis of key membrane phospholipids and is involved in malignant transformation in a large variety of human tumours. Active compounds against ChoK have been identified and proposed as antitumor agents. The ChoK inhibitory and antiproliferative activities of symmetrical bispyridinium and bisquinolinium compounds have been defined using quantitative structure–activity relationships (QSARs) and structural parameters. The design strategy followed in the development of the most active molecules is presented. The selective anticancer activity of these structures is also described. One promising anticancer compound has even entered clinical trials. Recently, ChoKα inhibitors have also been proposed as a novel therapeutic approach against parasites, rheumatoid arthritis, inflammatory processes, and pathogenic bacteria. The evidence for ChoKα as a novel drug target for approaches in precision medicine is discussed.

## 1. Introduction

Alteration of cell metabolism is a frequent event in human diseases [1]. In some instances are a necessary requirement, such as in the case of cancer onset and progression. A typical feature of cancer cells is an increased metabolic rate which supports unregulated growth and higher duplication rates. In keeping with this requirement, aerobic glycolysis, glutamine catabolism and lipid metabolism are frequently up-regulated in tumours [1]. As a key event, the pathways involved in the generation of the major phospholipids, such as cytidine diphosphate-choline (CDP-choline) and cytidine diphosphate-ethanolamine (CDP-ethanolamine), are frequently altered in human tumours. One of the enzymes that regulate these pathways is Choline kinase α (ChoKα). Evidence that ChoKα plays a critical role in many human diseases is increasingly being accumulated, and ChoKα has become the focus of a targeted therapeutic strategy [2].

As a consequence, small molecule inhibitors and small interfering RNA (siRNA) that interfere with the function of ChoKα have been designed. These reagents have proven to be effective and selective anticancer drugs [2]. We describe here how the most active antiproliferative and antitumoral agents against ChoK were designed by relying on quantitative structure–activity relationships (QSARs) and structural parameters. The initial framework of these inhibitors was based on symmetrical bispyridinium and bisquinolinium structures.

ChoKα inhibitors have also been proposed as a novel therapeutic approach against malaria, rheumatoid arthritis, pathogenic bacteria and inflammatory processes [3]. The aim of this review is to recount the history of the development of ChoKα inhibition therapies against human diseases and to summarize the most striking findings in this field. It is a fascinating journey from bench to the bedside. In addition, there are many paths yet to be fully explored in areas of illnesses other than cancer.

## 2. The ChoK Family in Humans

**Figure 1:**
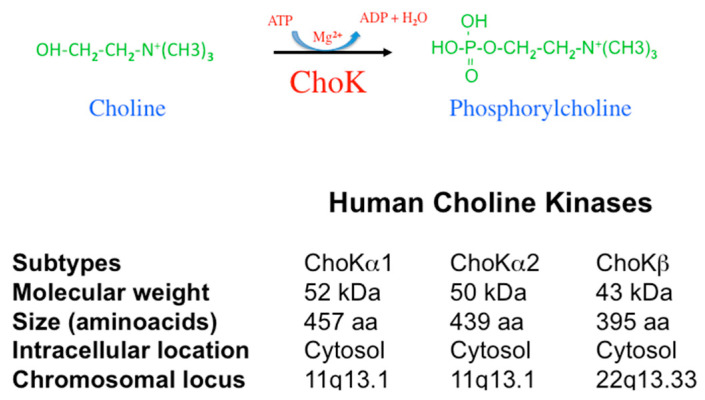
ChoK characteristics. ChoK catalyzes the phosphorylation of choline, rendering phosphorylcholine. This reaction requires Mg^2+^ and ATP. In humans, two distinct genes, CHKA and CHKB, code for the ChoK enzymes. The locus of these genes is indicated. CHKA generates ChoKα1 and ChoKα2 by differential splicing. Their molecular weight and size are included as well as their intracellular location.

In humans, two genes code for the enzymatic activity responsible for the generation of phosphorylcholine (*P*Cho) designated as CHKA and CHKB. Located in chromosomes 11q13.1 (CHKA) and 22q13.33 (CHKB), they code for proteins of ca. 50 kDa (ChoKα) and 45 kDa (ChoKβ, 395 amino acids) [4,5,6]. The CHKA gene originates by differential splicing ChoKα1 (52 kDa, 457 amino acids) and ChoKα2 (50 kDa, 439 amino acids) (Figure 1).

There is an overall sequence identity of 56% between ChoKα and ChoKβ proteins. Nevertheless, they seem to serve different metabolic and biological functions. ChoKα is essential for mouse [7] and plant [8] embryo development. A ChoKα knockout cannot be rescued by ChoKβ [7]. Meanwhile, ChoKβ is dispensable in mice and a ChoKβ Knockout (ChoKβ KO) leads to a non-lethal, rostrocaudal muscular dystrophy and forelimb deformity due to decreased levels of phosphatidylcholine [9] that can be rescued by ChoKα [10]. More recently, the role of ChoKβ in bone homeostasis in mice has been demonstrated [11]. This result is consistent with the fact that pharmacological inhibition of ChoK activity in human osteoblasts results in defective mineralization in vitro [12]. In humans, mutations in the CHKB gene lead to similar pathology [13]. Finally, in Arabidopsis, there are four Choline-Ethanolamine Kinase (CEK) genes related to choline (Cho) and ethanolamine (Etn) phosphorylation, with different roles in metabolism and development [14].

Additional enzymatic analyses revealed differential activity patterns for ChoKα and ChoKβ, ϕ υ ρ τ η ε ρ σ υ π π ο ρ τ ι ν γ different physiological roles. Although both have Cho kinase and Etn kinase activities under in vitro conditions, in whole cells assays they divert. ChoKα still has both activities but ChoKβ has only the ethanolamine kinase activity [15]. Furthermore, ChoKβ does not compensate for decreased phosphatidylcholine (PC) biosynthesis in ChoKα^+/−^ heterozygous mice. ChoKα production is sufficient to maintain normal PC levels in most tissues, but not in limb muscles of ChoKβ KO mice. Furthermore, phosphatidylethanolamine (PE) levels are unaffected in ChoKβ KO mice, a result which suggests that PE homeostasis is fully maintained when the ChoKα protein is intact [9]. Again, in Arabidopsis, substrate specificity for Cho and Etn are distinct for the four CEK enzymes [14] and resembles the situation described in mammalian cells [15].

The crystal structure of human ChoK demonstrates that this enzyme forms a dimer (DOI: 10.2210/pdb2CKQ/pdb; DOI: 10.2210/pdb5EQY/pdb) [16,17]. This structural feature, confirmed in enzymatic analysis may have important biochemical and biological consequences since different levels of enzymatic activity for homo- and heterodimers have been reported, with the ChoKα homodimer as the most active and the ChoKβ homodimer as the least active [18,19]. Furthermore, a balance of expression of the two isoforms may have a differential effect on the regulation of the cell cycle [18,19]. Dimerization ratios may therefore serve as a complex regulatory mechanism for enzyme function, though further work has to be done to clarify this system.

## 3. ChoKα in Cancer: A Promissing Therapeutic Target

ChoKα is overexpressed in a large diversity of human tumours including breast [20], lung [21,22,23], colorectal [21,24], bladder [25], prostate [21,26,27,28], ovary [29], endometrial [30], pancreas [31], liver [32,33,34,35], esophagus [36], and T-cell lymphoma [37]. ChoKα overexpression has also been reported in osteosarcoma tumour-derived cancer cells [12], Hepatitis B Virus (HBV)-induced hepatocarcinomas [38,39], breast, colon- and liver-derived cancer cells [40], glioblastoma- [41] and glioma-derived cell lines [42], pancreatic tumour-derived cell lines [31,43] and T-cell acute lymphoblastic leukemia (T-ALL) primary cells and commercial cell lines [44]. In addition, ChoKα expression levels appears to be a marker of cancer prognosis, aggressiveness or metastasis, in lung [22,23], breast [45,46], bladder [25], prostate [26,27], ovary [29], colorectal [24], esophageal squamous cell carcinomas [36], liver [34,35] and B-Cell lymphomas [47,48]. In breast cancer, it has been associated with ER^−^ status, increased invasiveness, and drug resistance [20,45,46,49,50].

Since ChoKα has an established role in the onset and progression of human cancers, this enzyme was proposed as a novel therapeutic target for cancer [2]. Therefore, a program for the design and synthesis of specific inhibitors (see Section 4 below) was established. siRNA attenuation of ChoK production was also used to support the proof of concept [51,52,53]. Thus, small molecules and specific siRNAs with potent antitumor activities both in vitro and in experimental animal models have been developed.

To further support the importance of ChoKα as a target for cancer therapy, ChoKα inhibition has been used in combinatorial regimes and showed a potent antitumor effect that synergizes with 5-FU in breast and colon cancer cells [54,55,56] and reverses resistance to TRAIL in colorectal [57] and ovarian cancer cells [58]. Similar synergic effects have been obtained with pancreatic ductal adenocarcinoma-derived cancer cells with gemcitabine, 5-FU or oxaliplatin [31], and for lung cancer cells with acid ceramidase inhibitors [59] or cisplatin (Lacal, unpublished).

PC synthesis is an absolute requirement of cancer cells in order to proliferate. Thus, inhibition of ChoKα has been shown to have therapeutic effects in a variety of tumor-derived cancer cell lines and tumor xenografts [2,3]. Blocking this metabolic pathway has been shown to be non-toxic to normal, primary cells as well as immortalized non-tumorigenic cells, implying that different responses are activated in tumorigenic and non-tumorigenic cells in different cell systems [60,61,62,63].

A large number of studies use magnetic resonance spectroscopy (MRS), high-resolution magic angle spinning MRS (HR-MAS MRS) or positron emission tomography (PET) to determine ChoK levels or their products as biomarkers in cancer diagnosis and prognosis have been reviewed recently. Therefore, although this is a very important field in development, these techniques will not be the subject of this review.

## 4. Design of ChoKα Inhibitors as a Precision Medicine Strategy

The cost of drug discovery is far too high to be led solely by trial and error. In computer-aided drug design, quantitative structure–activity relationship (QSAR) studies are an effective and useful approach. A QSAR study attempts to understand differences in the biological activities of a group of congeners in terms of molecular changes induced by substituent changes.

The most potent ChoK inhibitor known in the early 1990s was hemicholinium-3 (**1**, HC-3) with an IC_50_ for ChoK inhibition ex vivo of 500 μM using recombinant ChoK, and an IC_50_ against the human HT-29 cell line of 2.5 mM [64]. A high affinity paralyzing respiratory effect is induced by HC-3 [65], which is a competitive inhibitor of Cho transport. As a result, **1** cannot be used as an antiproliferative agent in vivo. Improving the potency and diminishing the toxicity of **1**, by changing its specificity and antiproliferative activity, was the first step in developing potential anticancer drugs that target ChoK. The ChoK inhibitory and antiproliferative activities of symmetrical bispyridinium and bisquinolinium compounds have been defined using quantitative structure–activity relationships (QSARs) and structural parameters. We will review this strategy in the following sections.

### 4.1. QSAR Studies between Ex Vivo ChoKα Inhibition and The Electronic Effects of Substituents at Position 4 of The Pyridinium Moieties

**Figure 2:**
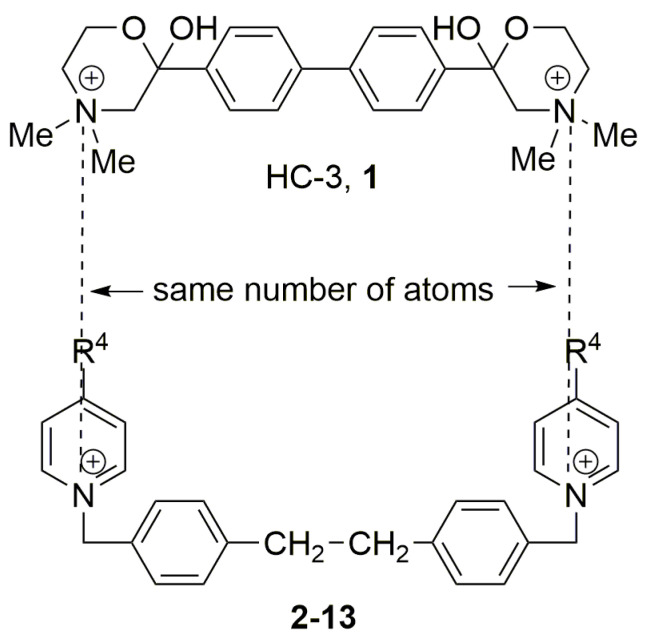
Molecular variations carried out from hemicholinium-3 (HC-3, **1**).

Two choline-like chains comprising quaternary groups are bound to the central biphenyl system in the HC-3 structure, forming oxazonium rings in solution [65]. The 1,4-oxazonium moieties of **1** were replaced with pyridinium rings with various substituents at position 4 in order to investigate their electronic effects. The substitution could be carried out at position 2 of the pyridinium ring, in which the possible electronic effects (inductive and mesomer) could also be operational; however, in the latter position, the sterical effect might have masked or altered the electronic effect. Therefore, in our earlier sudies we eliminated the possibility of substitution at the position adjacent to the N^+^. We have used the 1,2-ethylene(bisbenzyl) moiety as a linker to maintain the number of atoms between the two positively charged nitrogen atoms of **1** (Figure 2).

The compounds were screened in an ex vivo system using the human recombinant ChoK as a target. We determined the effect of our compounds on the ChoK enzymatic activity regardless of other confounding factors such as permeability into cells, special cellular conditions, intracellular modifications of compounds or compartmentalization. ChoK inhibitors were also tested on the HT-29 cell line to see how they affected cell proliferation in tumor cells [66]. This cell line was developed from a colon adenocarcinoma, one of the most common solid human cancers that are resistant to chemotherapy, making these cells suitable for the production of new antitumor drugs. The Ghose–Crippen modified atomic contribution method (ATOMIC5 option) [67] of the PALLAS 2.0 software [68] was used to measure the clog *P* (calculated log *P*) values of the bis-salts. π_spacer_ is the linker substituent constant and π_cat head_ is the –CH_2_–C_6_H_4_–(CH_2_)*_n_*–C_6_H_4_–CH_2_– group substituent constant, determined with the PALLAS 2.0 program [67] Ghose–Crippen modified atomic contribution method (ATOMIC5 option) [67].

The activity against the HT-29 cell line is, on average, higher than the activity against ChoK. Thus, the symmetrical bisquaternized salts may be affecting a different part of the pathway in this tumor cell line (see Table 1, Table 2 and Table 3).

All of the R^4^ substituents are either electron-releasing, neutral, or electron-withdrawing (–NMe_2_, –NH_2_, –CH_2_OH, –Me, –COOH, –C≡N). The electronic parameters for resonance effect (σ*_R_*) were published by Charton [69], and the following correlation Equation (1) emerges from data of Table 1 [64,72]:

*p*(IC_50_)_ex vivo_ = 3.92 (±0.00) − 0.92 (±0.06) σ*_R_* *n* = 5, *r* = 0.994, *s* = 0.052, *F*_1,3_ = 250.13 (significance at α < 0.001) (1)

where, *p*IC_50_ = −log IC_50_, bearing in mind that the higher the value of *p*IC_50_ the more potent is the compound, *n* is the number of compounds, *r* is the correlation coefficient, *s* is the standard deviation, *F* is the *F* ratio between the variances of observed and calculated activities, and data within parentheses are standard errors of estimate.

The inhibitory potency of the compounds is unrelated to their inductive effect [64], but is well correlated with their resonance effect. This is in line with traditional chemical principles. The positively charged ring nitrogen is in direct conjugation with R^4^. The higher the resonance effect of R^4^, the better the delocalization of the positive charge. Following these findings, researchers looked for a substituent with a higher electron-releasing effect than the –NH_2_ or –NMe_2_ groups. As a result, endocyclic amino groups like pyrrolidino, piperidino, and perhydroazepino may have a stronger electron-releasing effect. The *N*-methylanilino and the *N*-diallyl groups were also tested. Furthermore, when compared to the –NH_2_ and –NMe_2_ groups, their higher lipophilicities can aid in the antiproliferative activities of even more active compounds [70].

^13^C NMR spectroscopy was used to estimate the unknown σ*_R_* descriptors for the diallylamino, pyrrolidino, piperidino, perhydroazepino, and *N*-methylanilino moieties [71]. The totality of the ten compounds synthesized until then give rise to Equation (2):

*p*(IC_50_)_ex vivo_ = 3.92 (±0.11) − 1.09 (±0.15) σ*_R_* *n* = 10, *r* = 0.928, *s* = 0.181, *F*_1,8_ = 49.78 (significance at α < 0.001) (2)

**MN58b** is a member of the first generation of HC-3 derivatives that were used as a prototype for studying ChoKα effects in both normal and tumor cells [2].

### 4.2. Ex Vivo ChoK Inhibition and Clog P: QSAR Studies

We chose structures with the following characteristics to investigate the potential effect of lipophilicity on ChoK inhibition in ex vivo conditions [72]:

(a) Cationic heads with a “*zero electronic effect*”, i.e., hydrogen at position 4, that allow the positive charge to be dispersed to a large extent. We used unsubstituted quinolinium and isoquinolinium rings to achieve this.
(b) Aralkyl spacers with different number of methylene groups.

ChoK inhibition activity is found to correlate with lipophilicity for these compounds, as shown in Equation (3):

*p*(IC_50_)_ex vivo_ = 4.83 (±0.09) + 0.81 (±0.15) clog *P* *n* = 7, *r* = 0.922, *s* = 0.151, *F*_1,5_ = 28.57 (significance at α < 0.005) (3)

On the basis of Equation (3), we propose that hydrophobic interactions between the bisalts **13**–**20** and ChoK can occur. The QSAR greatest contribution is that it has provided a comprehensive and reasonably complete quantitative understanding of the role of hydrophobicity in drug action [73]. Hydrophobicity is related not only to absorption and distribution but also to the interactions with ChoK active site. Apart from electrostatic interactions, it appears that the cationic heads and spacers between the two positive nitrogen atoms, hydrophobilically bind to the enzyme more strongly. Non-covalent interactions in aqueous solution are dominated by hydrophobic interactions. At hydrophobic surfaces, loosely connected water molecules have a degree order and are therefore in an undesirable entropic degree. The interaction of a drug hydrophobic areas with its binding site releases the organized water molecules, resulting in an increase in entropy. The planar quinolinium and isoquinolinium cations have been shown to bind to artificial receptors, with aromatic rings, more strongly than the alkylammonium cations [74].

### 4.3. Combining The Electronic and The Lipophilic Effects in The Same Molecules

The electron-releasing potential of the substituent at position 4 of the heteroaromatic cationic head (Table 1) and the lipophilicity of the bissalts (Table 2) had a significant effect in ChoK inhibition. Therefore, in order to improve the inhibitory efficacy, we combined these two properties in the same molecule and prepared compounds **21** and **22** (Table 3). The -NH_2_ group is an electron-releasing group that should be strong, while the pivaloylamino moiety should be weak. We used the corresponding value of the very similar acetamido group instead (σ*_R_* = −0.35) [69] because the latter σ*_R_* value was unavailable. The results met our standards, so we tried to link the ChoK inhibitory potency with the two descriptors, resulting Equation (4), in which all the compounds of Table 1, Table 2 and Table 3 were included:

*p*(IC_50_)_ex vivo_ = 4.44 − 0.73 (±0.14) σ*_R_* + 0.12 (±0.04) clog *P* *n* = 19, *r* = 0.836, *s* = 0.241, *F*_2,16_ = 18.61 (significance at α < 0.001) (4)

Our QSAR studies revealed that the efficacy of inhibitors is determined by π at a particular location on the molecules rather than the *overall* log *P*. Equation (5) suits very well with the use of these site-specific π parameters:

*p*(IC_50_)_ex vivo_ = 0.55 − 1.04 (±0.13) σ*_R_* + 0.63 (±0.15) π_spacer_ + 0.30 (±0.08) π_cat head_ *n* = 19, *r* = 0.917, *s* = 0.181, *F*_3,15_ = 26.46 (significance at α < 0.001) (5)

In order to obtain Equation (5), we had to accept that for compounds **2**–**12**, the π_cat head_ was equal to zero (π_2H_ due to the H-2 and H-3 protons of the pyridinium moiety), while for bisquinolinium and bisisoquinolinium structures **13**–**22**, π_cat head_ is 1.27. π_spacer_ is the –CH_2_–C_6_H_4_–(CH_2_)*_n_*–C_6_H_4_–CH_2_– group substituent constant, which ranges between 5.14 (*n* = 0 of the spacer) and 6.28 (*n* = 3 of the spacer). The fact that π_spacer_ has a higher coefficient and values than π_cat head_ leads to the inference that the former is much more significant than the latter in terms of hydrophobicity. Finally, the positive π-term coefficients indicate that hydrophobic moieties and electron-donating groups support ChoK inhibitory action, at least within the context of spacers and heteroaromatic rings used.

### 4.4. Influence on The Antiproliferative Activity Against The HT-29 Cancerous Cell Line

The final research focused on two aspects: (a) the impact that a variation of the linker that links the quinolinium cations with electron-releasing groups at position 4 with other different groups at positions 3, 7, and 8 of the heterocycle (compounds **23**–**63**) would have on the ex vivo human inhibitory activity; and (b) the impact of the factors that regulate the antiproliferative properties of such compounds [66].

Table 4, Table 5 and Table 6 show the biological results of compounds **23**–**63**, which correspond to series **A**, **B**, and **C**, as a function of the group at position 7 of the quinolinium ring.

Lipophilicity, molar volume, and steric bulk are all factors that influence molar refractivity (MR) [75]. To get rational values for the regression coefficients of the resulting QSAR equations, the MR values are usually scaled by a factor of 0.1. With the support of 3D structures, the importance of MR in QSAR equations of certain ligand–enzyme interactions were interpreted. These studies revealed that π-modeled substituents bind in a hydrophobic space. Interactions between these substituents with a polar surface will give rise to a positive sign of MR in a QSAR equation [76,77], while a negative sign or a nonlinear relationship indicates a limited area or steric hindrance at this binding site [75].

Equation (6) was obtained after taking into account the volume effects (MR_8_) (the subscript refers to the location of the substituent), the measured global lipophilicity (clog *P*), the linker substituent constant, and the electronic parameters (σ*_R_*) of the R^4^ substituent for antiproliferative activity:

*p*(IC_50_)_HT-29_ = − 2.66 − 0.03 (± 0.00) MR_8_^2^ + 0.10 (± 0.02) clog *P* + 1.05 (± 0.31)π_linker_ − 3.73 (± 0.71) σ*_R_* *n* = 40 (series **A**, **B** and **C**), *r* = 0.920, *s* = 0.223, *F*_4,35_ = 47.856, α < 0.001 (6)

The following points should be illustrated from Equation (6): (a) Since the coefficient of MR_8_ is a squared negative expression, the existence of a methyl group at position 8 is detrimental to antiproliferative activity; thus, a hydrogen atom at this position is preferable because it has a lower MR value; (b) Lipophilicity contributes to the antiproliferative activity in two ways: on the one hand, it has a global contribution (clog *P*), and on the other, it has a particular contribution at a specific location on the molecules (π_linker_). Since both descriptors are orthogonal, their inclusion in the QSAR equation is justified. Since π_linker_ has a higher relative contribution in Equation (6) than clog *P*, it is possible that favorable hydrophobic interactions between the enzyme and the linker modulate the inhibitor-ChoK coupling. While raising the global lipophilicity and the linker lipophilicity would increase antiproliferative activity, solubility was the reason for limiting the spacers to 3,3′-, 4,4′-biphenyl, and 4,4′-bibenzyl moieties.

Equation (7) is obtained when the experimental *p*(IC_50_)_HT-29_ values are compared with the theoretical ones determined by Equation (7):

*p*(IC_50_)_HT-29 exptl_ = − 0.35 + 1.05 (±0.08) *p*(IC_50_)_HT-29 theoret_ *n* = 40, *r* = 0.916, *s* = 0.220, *F*_1,38_ = 198.854 (significance at α < 0.001) (7)

Since it obtained the best experimental results, compound **RSM-932A** was selected for further study. It acts similarly to **MN58b** in vitro by inducing cell cycle arrest in nontumorigenic cells and apoptosis in tumor cells [60,61,62,63]. **RSM-932A** has no detectable toxicity in mice at highly effective doses that lead 77 percent tumor growth inhibition in in vivo conditions [78]. Indeed, **RSM-932A** (also known as **TCD-717**) was the first ChoKα inhibitor to be studied in humans, in a phase I clinical trial [79].

### 4.5. Other Symmetrical and Unsymmetrical ChoKα Inhibitors

**Figure 3:**
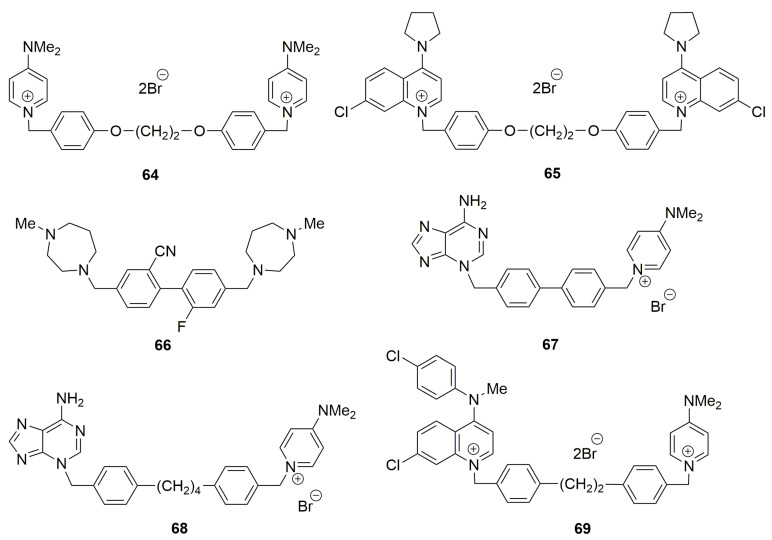
Several symmetrical bis-charged (**64**, **65** [80]), symmetrical uncharged (**66** [81]), unsymmetrical mono-charged ChoKα inhibitors (**67**, **68** [82]) and bis-charged ChoKα inhibitors (**69** [83]).

Other modifications have been carried out to give rise to newly designed structures **64**–**68** (Figure 3).

Symmetrical bis-charged structures containing minor modifications in the spacer have been described [80]. Unfortunately, because of their structural homology to choline, double positively charged chemotypes are correlated with substantial toxicity due to off-target activities [81]. The lead series described by Zech et al. [81], on the other hand, relies heavily on basic amines found in methyldiazepanes to bind to the choline site. A primary hydrogen bond is added by the protonated 1’ nitrogen. It is important to remember that depending on local conditions like pH, the methyldiazepane moiety may be transiently protonated or present as a free base. Furthermore, diazepane is used in a wide variety of licensed medications, mainly to boost solubility or ADME parameters. In most cases, this moiety does not pose a significant toxicity risk. Compound **68** induced apoptosis at low μM concentrations in the ChoKα expressing line MDA-MB-415.

From their synthesis to the molecular basis of their binding mode, a review in the field of ChoK inhibitors was published [82]. The first library of asymmetric bispyridinium derivatives was reported by Rubio-Ruiz et al. [82]. To investigate potential new binding modes to the enzyme. 4(4-Chloro-*N*-methylanilino)pyridinium, 1-benzyl-4-(dimethylamino)pyridinium, and 4-pyrrolidinopyridinium fragments were combined in these structures, with both positively charged moieties linked with variable linkers. The plasticity of the choline binding site, the discovery of new exploitable binding sites, and the allosteric properties of this enzyme are all emphasized as strategies for developing inhibitors and their selectivity on ChoKα over ChoKβ. Rubbini et al. published a new class of asymmetrical pyridinium/quinolinium derivatives developed and designed based on drug optimization. Compound **69** does not promote apoptosis and does not increase the cleavage of PARP, indicative of caspase activation. It induces a cell-cycle arrest in G1 Phase and induces senescence in MDA-MB-231 [83].

## 5. ChoKIs Mechanism of Action

The implication of ChoKα in a diversity of human cancers is well established. Its lack of expression is lethal, implying that its function is essential [7], and its inhibition leads to either cell arrest in non-tumorigenic cells, or cell death in tumor cells [2,3,60,61,62,63]. Since ChoKs use choline and ATP as substrates of the enzymatic reaction, inhibition of ChoKα activity by direct blockage of the Cho or ATP-binding pockets could be a specific and effective strategy to block cancer cell proliferation. A number of ChoKIs have been designed to achieve this goal [82].

However, this strategy has been questioned since V-11-0711, a potent inhibitor of ChoKα that affect only its catalytic activity, is not able to induce cell death in breast tumour cells. Therefore, a non-catalytic function of ChoKα has been suggested [84,85]. In keeping with this, a balance between ChoKα and ChoKβ may be required for the appropriate regulation of cell proliferation and reduction of the protein levels of ChoKα may be required in some cell systems for induction of cell death [19]. Furthermore, a chaperone activity has been reported for ChoKα [86,87]. If this is correct, effective ChoKαIs must be able to simultaneously reduce ChoKα enzymatic activity and intracellular protein levels.

The enzymatic activity of ChoKα follows a ping-pong mechanism with an intermediary phosphorylated conformation that can be used for its inhibition without affecting ATP and Cho binding by a steric interaction [88,89]. In keeping with this, **RSM932A/TCD717** has a unique mechanism of action, different from any other ChoKαI so far described. Its effect is not competitive with choline or ATP [88] since it does not bind directly in the choline or ATP pockets as do previously characterized ChoKαIs, but rather in a proximal but novel location near the surface of the enzyme [90]. This characteristic renders a rather unique mechanism of action that potentiates its specificity and potent antitumor activity [78].

Inhibitors such as **RSM-932A** or specific siRNAs with a drastic reduction of both the levels of ChoKα and its product, phosphorylcholine, show a drastic and selective induction of cell death in a large variety of tumor cells. Additionally, it has been suggested that **RSM932A** induces a conformational change that expose the unfolded structure of the enzyme to endogenous proteases resulting is a drastic reduction of the ChoKα protein levels [62,88]. Consistent with this interpretation, inhibition of ChoKα activity with no significant reduction of its protein levels may not be sufficient to trigger cell death in tumor cells [84,85]. However, in glioblastoma cells, inhibition of ChoKα by **V-11-0711** is sufficient to significantly reduce cell viability, invasiveness and clonogenicity. Furthermore, this antitumoral effect was related to inhibition of the expression of EMT associated genes mediated by ChoKα, and was synergistic with temozolomide [91]. Thus, the effect on cell viability by ChoKIs may be cell type dependent.

Several studies have looked into the implications of interfering with ChoK function, which may clarify how ChoK specific inhibitors (ChoKIs) and ChoK silencing with specific siRNA operate. ChoK inhibition through pharmacological or siRNA approaches cause a variety of effects such as loss of mitochondrial potential and cytochrome c release [58], increased ceramide output [61], ER stress [62], unfold protein response (UPR) [62], and ROS homeostasis via glutathione levels [92]. Similar effects on ER stress have been reported in Arabidopsis [93].

Decreased mitochondria function by pharmacological inhibition of ChoKα has been associated with a reduction of citrate synthase expression and AMPK activation. These effects are also related to an increase in glucose and acetate uptake in an attempt to overcome the metabolic stress. The final outcome is the induction of cell death including apoptosis or necrosis with an exquisite specificity towards cancer cells [60,61,62,63,91].

The implication of ChoKβ in cancer is still not resolved. Genetic evidence has clearly demonstrated a role of ChoKβ in muscular dystrophy [9,10,11,12,13]. Furthermore, ChoKβ inhibition by siRNA has no detrimental effect in cell viability, maybe because ChoKα can supplement its absence as a redundant metabolic enzyme [9]. However, we cannot exclude the possibility of a role of ChoKβ in the modulation of the activity of ChoKα in different tissues and therefore can still play a critical role in the onset and progression of tumours [18,19].

## 6. ChoKα in Drug Resistance

Drug resistance, one of the major burdens in anticancer research, has been also investigated in the ChoK pathway. In NSCLC-derived cells, resistance to ChoKαIs is overcome by overexpression of acid ceramidase (ASAH1) in keeping with the known mechanism of action of ChoKαIs of inducing increased levels of ceramides [59,61] and allows the identification of tumours resistant to treatment with ChoKαIs. Acquisition of resistance to ChoKαIs has also being found in pancreatic [31] and breast cancer cells (Lacal et al., unpublished). In B-Cell lymphomas, resistance to lysine deacetylase inhibitors (KDACI) is linked to increased ChoKα activity and can be overcome by treatment with ChoKαIs (48). Similar effects have been reported in TRAIL-resistant colorectal [57] and ovarian tumours [58] where ChoKαIs can restore sensitivity to TRAIL.

Differences in the response of normal, non-tumorigenic and cancer cells have been unveiled. Thus, in breast, lung and colon-derived tumor cells, a drastic alteration of the levels of expression of proteins involved in the regulation of endoplasmic reticulum (ER) stress and the unfold protein response (UPR) takes place as a consequence of ChoKα inhibition [62]. A completely different scenario is observed in non-tumorigenic cells, where a transient and attenuated ER stress response is observed. These alterations in protein expression occur in parallel with a drastic reduction in cyclin D1, RB and E2F1α in cancer cells which is not observed in non-tumorigenic cells [62]. Thus, maintenance of the RB-E2F1α complex in non-tumorigenic cells after ChoKα inhibition induces arrest in G0/G1 but not cell death [62]. These results are consistent with many studies where a differential effect was observed in cancer versus non-tumorigenic cells [2,3]. Additionally, in Arabinopsis, a similar role for the CEK1 enzyme has been proposed for the management of ER stress [92].

Likewise, specific interference with ChoKα siRNA drives cancer cells to apoptotic death [2,3,51,52,53,55]. By contrast the specific ChoKα inhibitor **V-11-0711** (IC_50_ = 20 nM), causes reversible growth arrest [84], similar to what is observed in non-tumorigenic cells with other inhibitors [60,61,93]. These observations suggest that ChoKα plays a role in cancer cell survival that is outside the PC synthesis pathway [84] in keeping with abundant information that links ChoKα with both metabolic and signal transduction functions. These results are further supported by transcriptome analysis in cancer cells since inhibition of ChoKα induce alteration of expression levels of genes involved in cell cycle regulation, apoptosis, and nucleotide metabolism [94,95,96,97].

## 7. ChoKs, More Than Metabolism Enzymes?

In addition to all the reported effects on cell metabolism, interference with ChoKα activity and levels has also an effect in signal transduction pathways. ChoKα has been shown to be phosphorylated by c-Src in breast cancer cells and as a consequence, it associates with the EGFR and is translocated to the plasma membrane [98]. Phosphorylation takes place at Y197 and Y333 and increases its activity by 1.40- to 1.68-fold when ChoKα is expressed along with c-Src or EGFR, respectively, and about 2.5-fold when co-overexpressed with both EGFR and c-Src [98]. Phosphorylation of ChoKα seems to correlate with a higher cell proliferation rate. Recently, this interaction has been mapped at the SH3 domain of c-Src and the poly-proline region N-terminal of ChoKα [99]. The relationship of ChoKα and EGFR, has also been reported in lung [100] and liver tumors [35] and has been associated with resistance to EGFR inhibitors [35]. In prostate cancer, ChoKα acts as a chaperone for the androgen receptor (AR), elucidates a feed-forward signalling loop that maintains ChoKα expression and as a consequence reinforces AR signaling activity. This chaperone function confers a growth advantage to cancer cells where ChoKα is overexpressed [89,90].

By contrast, ChoKβ activity is regulated by PKA at residues Ser 39 and Ser 40, a process that depends on cAMP levels [101]. Phosphorylation of ChoKβ increases its catalytic efficacy for choline and ATP but not to ethanolamine, and results in an increased sensitivity to HC-3 inhibition. This could be consistent with that phosphorylation of ChoKβ may be a mechanism to regulate PC and PE synthesis and that phosphorylation could modulate the balance of both isoenzymes as previously described [19]. Additionally, a differential phosphorylation by c-Src and PKA suggests that both isoforms could be implicated in carcinogenesis although by alternative mechanisms and in different organs.

Regulation of ChoKs is therefore similar to other eukaryotic protein kinases (ePKs) and follows phosphorylation patterns, in keeping with their proposed common ancestor and with an early eukaryotic origin. In fact, both ePKs and ChoKs have been suggested to have played an important role in early evolution of highly complex eukaryotic cells [102].

ERK and AKT phosphorylation are reduced by either pharmacological inhibition or siRNA silencing of ChoKα in breast and T-cell lymphoma cancer cells [103,104,105]. Finally, ChoKα is regulated by PI3K and Ral-GDS, two direct effectors of the Ras oncoprotein [106].

MYC and HIF1 have been involved in the regulation of ChoKα expression [37,107,108], suggesting that overexpression of ChoKα results from the acidification typical of solid tumors. HIF1 is modulated in gliomas by 2-HG (2-hydroxyglutarate), the product of IDH1 (isocitrate dehydrogenase 1), resulting in a reduction in ChoK and EtnK activity [109].

ChoKα has been involved in neuronal differentiation [110], a process mediated by CCAAT/Enhancer binding Protein-β (C/EBPβ) through the CHKA promoter and the involvement of the ERK1/2 pathway and KDM2B (Lysine Demethylase 2B) as a repressor [111,112]. In keeping with this observation, a relationship with Parkinson’s disease (PD) has been recently suggested for ChoKα by modulation of Daf-16 location and control of the lipid content [113]. Finally, ChoKα interacts with the nonstructural protein 5A (NS5A) and phosphatidylinositol-4-kinase III (PI4KIII), modulating hepatitis C virus (HCV) replication through a mechanism that involves the endoplasmic reticulum [114,115]. Inhibition of ChoKα has therefore a potential antiviral function.

ChoK dysfunction has recently been linked to a variety of other human diseases. ChoKα deficiency is lethal during early embryo development in mice and Arabidopsis [7,8], while ChoKβ is dispensable for embryo viability but required for proper forelimb development [9,10,11,12,13]. Differential expression patterns [116,117], mitochondrial activity [118], and neuromuscular junctions [119] are all effects of ChoKβ deficiency in mice. Inactivating mutations in the CHKB gene have been linked to human muscular dystrophy and myopathy in several recent studies [11,120,121,122,123,124,125,126,127,128]. Since a lack of ChoKβ activity causes muscular dystrophy, any intervention that restores ChoKβ activity can help to restore normal muscle development in this pathological situation.

Regulation of both CHKA and CHKB genes have been reported to follow distinct patterns. Thus, the ChoKβ is regulated along with the carnitine palmitoyltransferase 1B (CPT1B) gene and it is involved in the modulation of brown adipose tissue to generate heat [129]. Curiously, ChoKα is regulated in a robust circadian expression pattern in the liver and adrenal glands [130]. These findings reinforce the idea of differential physiological roles for both isoenzymes.

Searching for mechanisms that will allow to control ChoK functions, it is of interest to remark that two recent reports involve miRNA species in a network of regulatory processes to control ChoKα levels and function. It has been shown that miR-367-3p targets the 3’-UTR of the *chka* mRNA transcript with strong affinity in MCF7 breast cancer cells and, as a consequence, represses its expression [131]. In the lung cancer cell lines H1355 and A549, miR-1-3p regulates the EGFR/MAPK/ChoKα signaling pathway through modulation of the expression of *FAM83A* (Family with sequence similarity 83, member A) [100] a gene involved in the onset of lung adenocarcinoma [132]. These interesting findings open the possibility to explore alternative strategies for the control of ChoKα levels and activity as therapeutic approaches.

## 8. ChoKs and ChoKIs of Protozoal Parasites

Roughly 15% of the world population is affected at some point in their lives by an illness caused by a protozoan parasite. In the developing world, this figure increases to 65%. The available therapies for many of these types of protozoal infections are limited, inadequate, and increasingly useless as resistant strains emerge. At the same time, market logic ensures that research and development into novel drug therapies for treating many of these parasites is limited. Therefore, research into novel drug targets such as choline kinase is of paramount importance [133].

### 8.1. Plasmodium falciparum and ChoK

*Plasmodium falciparum* is a parasite which is the causative agent of malaria, which is transmitted by mosquitoes. Developing alternatives for the treatment of malaria is extremely critical: new drug-resistant strains of *P. falciparum* continuously appear. This includes strains resistant to artemisinin, the current frontline drug of choice in endemic regions [134]. Novel drug targets must be established and thoroughly researched, and drug discovery programs need to focus on discovering inhibitors for these novel target. One relatively novel drug target is choline kinase, an enzyme in the pathway leading to the production of Phosphatidylcholine (PC) and phosphatidylethanolamine (PE).

PC and PE are two essential and highly represented phospholipids in found in the membranes of *P. falciparum* (40% and 35% of total phospholipids, respectively) [135]. The essential nature of these phospholipids means that the pathways responsible for their synthesis can be disrupted to eliminate this parasite [136]. In *P. falciparum,* as in other eukaryotes, PC and PE are synthesized from choline (Cho) or ethanolamine (Etn), respectively, via the Kennedy pathway. The first enzyme in this pathway in *P. falciparum* is choline kinase (pfChoK), which functions both as a choline kinase and an ethanolamine kinase. Importantly, *pf-*ChoK is more highly expressed in growth phases of *P. falciparum* [137]. Inhibition of pf-ChoK affects the parasite’s viability in in vitro and mouse models of malaria [88,138]. Therefore, pfChoK is a natural target for inhibition.

The first pfChoKI, hexadecyltrimethylammonium bromid (HBA), was reported in 2007. HBA significantly reduces *P*Cho synthesis (by 57% at 10 µM) and parasite growth (81% at 20 µM) and reduces parasitemia by 50% in mice with doses of 5 mg/kg [138].

**Figure 4:**
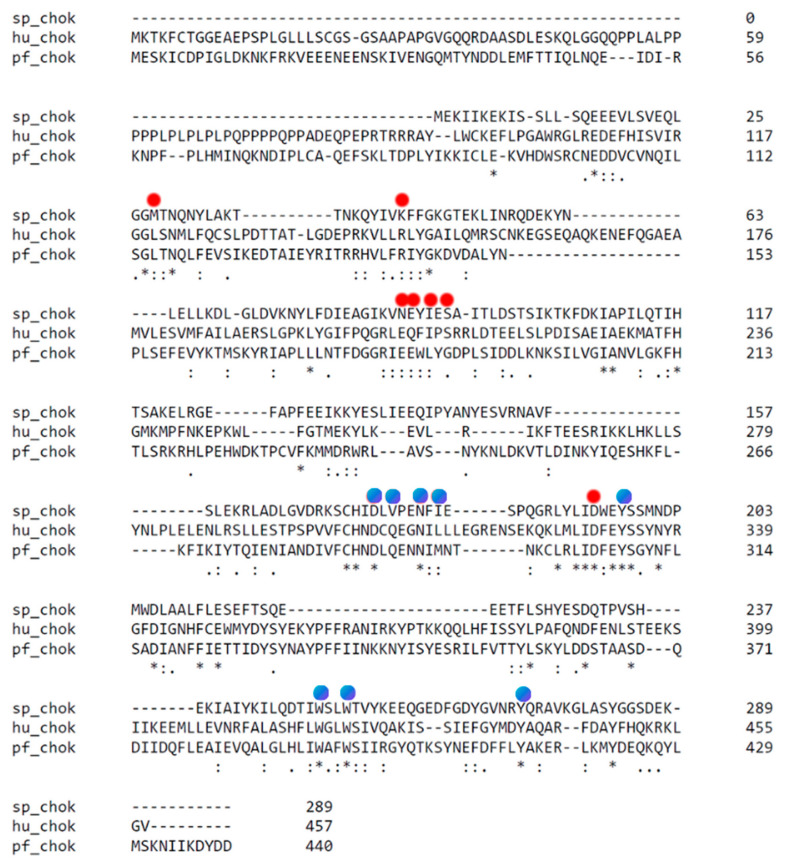
Alignment of the primary sequences of hChoK, pf-Chok, and sChok using the alignment tool ClustalW. Stars indicate conserved residues and the positions of the choline kinase binding residues of hChoK are marked with a blue circle. The ATP binding site residues are marked with a red circle. While there is poor overall alignment of sequences, there is strong conservation in the areas of the ATP and choline binding site, as can be seen by the number of residues that are either conserved or semiconserved.

**Figure 5:**
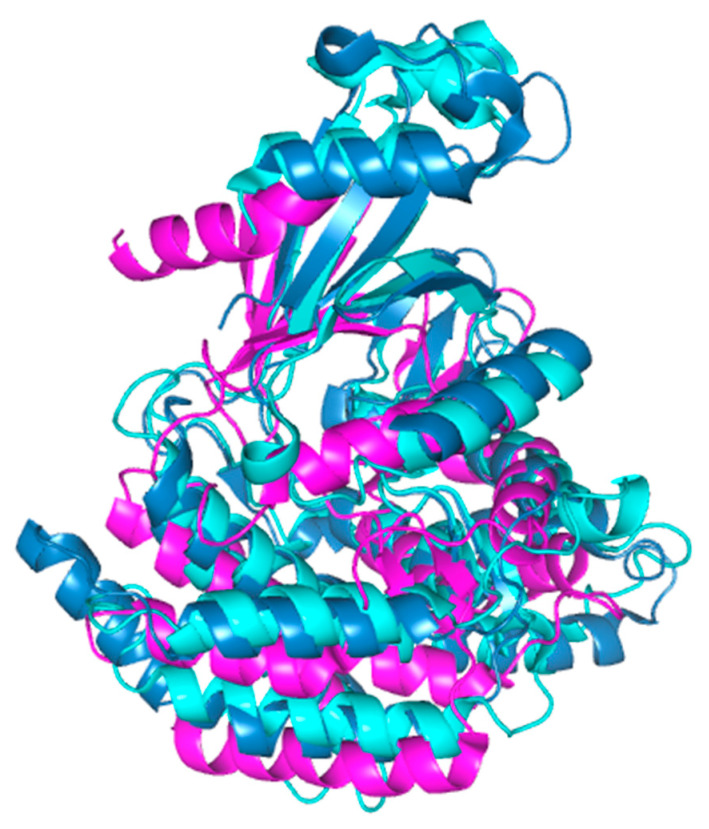
Alignment of the crystal structures of hChok (blue, RCSB accession #2CKO, dimer); pfChok (cyan RCSB accession #6YXS [140], monomer); and sChoK (purple. RCSB accession 4R77, monomer). The basic N-terminal and C-terminal domains are shown to be generally conserved. Alignment and figure generation carried out with the PyMol Package.

Some promising inhibitors have been discovered by the repurposing of hChoK inhibitors [88,139]. This repurposing strategy is effective because the primary structure of the active site of pf-ChoK (Figure 4), as well as the tertiary structure (Figure 5), are highly conserved with respect to hChoK [140]. For example, hChoK inhibitors **MN58b** and **RSM-932A** inhibit pfChoK in the micromolar range [88]. These inhibitors also prevent growth of *P. falciparum* at the low nanomolar range. In addition, they are effective against both drug-sensitive and drug-resistant strains. **MN58b** and **RSM-932** also prevent intraerythrocytic development, which affects parasite egress and invasion. **RSM-932A** has an uncompetitive mechanism with respect to choline and ATP while **MN58b** has a competitive mechanism with respect to both substrates.

Repurposed hChoK inhibitors which selectively inhibited the ethanolamine kinase function of pf-ChoK were also discovered. These blocked the growth of parasites at IC_50_s in the low nanomolar range. These inhibitors also caused a concomitant drop in cellular PE, leading to cell death [139].

Bisquinolinium bromide salt derivatives were discovered via high-throughput compound screening. These inhibitors were found to have affect *P. falciparum* growth at IC_50_s < 1µM and had IC_50_s against pf-ChoK in the low µM [141].

### 8.2. Leishmania infantum and Choline Kinase

*Leishmania infantum* is the causative agent of Leishmaniasis, a disease found in the tropics and subtropics and transmitted by phlebotomine sand fly. Leishmaniasis affects nearly 1 million people a year [142]. There has been an initial study whether or not ChoK inhibition is an effective strategy for eliminating *L, infantum* parasites. A number of quaternary ammonium salts were screened, however the most effective inhibitor, *(N-*iodomethyl-*N*,*N-*dimethyl*-N-(*6,6-diphenylhex-5-en-1-yl) ammonium iodide, only inhibited liChoK in the mM range. Therefore, more work needs to be carried out to discover more powerful inhibitors against liChoK and to assess their ability to kill the parasite [143].

### 8.3. Trypanosoma brucei and Choline Kinase

*Trypanosoma brucei* is the causative agent of Human African Trypanosomiasis also known as Africn sleeping sickness. This parasite is transmitted by the Tsetse fly. Several hundred thousand sub-Saharan Africans are infected yearly with this parasite, leading to 10,000 deaths. This parasite produces a single choline kinase isoform (tbChoK) [144]. Conditional knockouts tbChoK has demonstrated that this enzyme is critical for cell growth [145]. The only available treatments are often toxic and drug resistant strains have emerged [146]. To date, we have identified a single drug discovery study which utilized fragment based screening against tbChoK to identify leads that were effective against *T. brucei* at *IC_50_s* in the low µM [145]. These results suggest that tbChoK is a promising drug target.

## 9. Bacterial Pathogens and Their ChoKs and ChoKIs

The emergence of bacterial pathogens that are resistant to current antibiotic therapies underlines the need for continuous research into alternative therapies and the discovery of novel drug targets. A promising strategy is to exploit information regarding drug targets in eukaryotic systems and explore their analogs in bacterial systems. This is an ideal, cost-effective, and efficient strategy, because the same therapies that were developed at considerable expense in eukaryotic systems can be repurposed as antibiotics and antimicrobials. Promising examples of this strategy are bacterial pathways of which ChoK is a part. These pathways are best characterized in the Gram-positive *Streptococcus pneumoniae* and Gram-negative *Haemophilius influenzae.*

### 9.1. Streptococcus pneumoniae and Choline Kinase

**Figure 6:**
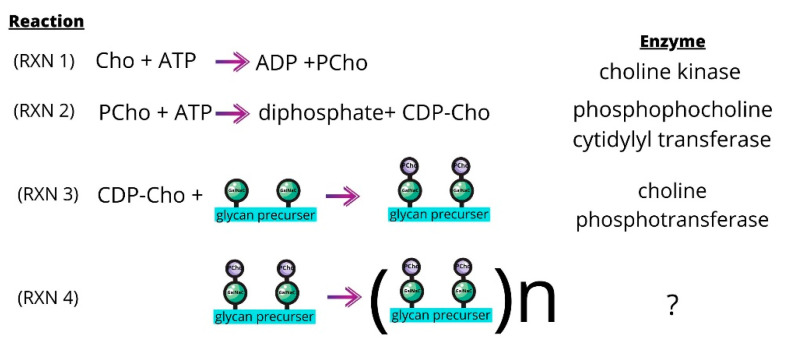
The sChok pathway and how it fits into the overall process of teichoic acid assembly. PCho molecules (purple) are transferred from CDP-Cho to the GalNac residues (green) of glycan precursors (RXN 3). The glycan precursors then assemble to form pre-teichoic acids (RXN 4), which become either LTA or CTA molecules depending on their final destination. LTA and CTA are embedded in the membrane or cell wall, respectively.

Choline is an essential nutrient for *S. pneumoniae* [147] and the choline kinase of *S. pneumoniae* (sChoK) is an essential enzyme [148]. This pathogen meets its needs for this metabolite in part by scavenging choline molecules from host cells using the Pce phosphodiesterase [149]. As in eukaryotes, the ChoK of *S. pneumoniae* (sChoK) phosphorylates choline (Cho) into phosphorylcholine (*P*Cho). However, the subsequent metabolic pathways diverge from the Kennedy pathway: *P*Cho is utilized in the pathways responsible for the synthesis of bacterial teichoic acids (Figure 6).

These are lipoteichoic acid (LTA) and cell wall teichoic acid (CTA) [150,151]. The LicC cytidylyl transferase catalyzes the production of CDP-choline from *P*Cho (Figure 6, RXN 2). The LicD1 and LicD2 *P*Cho transferases transfer the *P*Cho to the *N*-acetylgalactosamine (GalNac) residues found on pre-teichoic acid glycan precursors (Figure 6, RXN 3) [152].

The precursers are then assembled by an uncharacterized enzyme into variably sized pre-teichoic acid molecules, (Figure 6, RXN 4) [152]. Pre-teichoic acid transport across the cell membrane then mediated by the teichoic acid flippase TacF to become either LTA or CTA. LTA and CTA are chemically similar, with the difference that LTA is embedded in the cell membrane via a glycolipid lipid anchor through the action of the TacL ligase [153], and CTA is covalently attached to the cell wall via peptidoglycan molecules through the action of LCP phosphotransferases [145]. Meanwhile LTA is a known virulence factor and the LTA synthesis pathway has demonstrated to be a source of drug targets [154].

Significantly, LidD2 knockouts of *S. pneumoniae* diminish virulence due to limitations in the number of *P*Cho containing teichoic acids found on the cell surface [155]. *P*Cho decoration of the cell surface is required for normal cell-functioning because the Cho moiety anchors choline binding proteins (CBPs) such as murein hydrolases LytA and LytaAB, which are critical factors for cell division [156]. In addition, it anchors the choline binding protein A (CBPA) which is a determinant of virulence [157]. Many other CBPs are involved in colonization and even sepsis [158]. Therefore, the choline metabolic pathways, of which sChoK is an integral part, play an important role in *S. pneumoniae* growth and invasion.

Disrupting the teichoic acid production pathway can also disrupt *S. pneumonaie* cell growth. Therefore, it follows that disruption of any element of this pathway, such as sChoK, is a promising therapeutic avenue to follow. In addition, the same tools used the to inhibit eukaryotic ChoKs are likely to be effective against sChoK, particularly those know to interact with the substrate binding sites. This is because the choline and ATP sites of sChoks are highly conserved with respect to eukaryotic isoforms (Figure 4) as are the tertiary structures.

With this rationale in mind, the well-known choline analog and competitive hChoKI was studied and found to inhibit sChoK and cell growth with a high IC_50_ (in the mM range). This was also found to disrupt lipoteichoic acid production, which was consistent with the cell wall deformations that were observed by scanning electron microscopy. This established sChoK as a putative drug target [159,160,161]. Later, the hChoK inhibitors **MN58b** and **RSM-932A** were assayed and found to inhibit both sChoK activity and *S. pneumoniae* cell growth at moderate and low μM concentrations, to modulate lipoteichoic acid production and assembly, and to affect the cell wall. Interestingly, in the case of in vitro studies with purified sChoK, **MN58** functions as a competitive inhibitor against both choline and ATP, while **RSM932A** functions as a competitive and non-competitive inhibitor against choline and ATP, respectively. This result against **RSM932A** is in sharp contrast to the observed uncompetitive behavior of this drug in the case of pf-ChoK. This indicates that there are likely enough differences in the largely conserved active sites of the different ChoKs to modulate ChoKI behavior. This promising outcome suggests that it will likely be possible to rationally design ChoKI that are effective but selective between the different isoforms.

### 9.2. Haemophilius influenzae ChoK

The choline kinase of *Haemophilius influenzae* (hiChoK) is another promising target to explore for the design of inhibitors against this pathogen. Cho is not a nutritional requirement in the case of the Gram-negative *H. influenzae*, as it is in *S. pneumoniae*. Nevertheless, this pathogen does uptake this metabolite from its surroundings [162], including from host cells [163] *H. influenzae* produces *P*Cho and uses it to decorate its lipopolysaccharides (LPS). *P*Cho containing LPS molecules mediate the interactions between the pathogen and host and help the pathogen avoid host immune responses [149]. By mimicking the phosphatidylcholine of eukaryotic host cells, these LPS molecules shield the pathogen from attack from host cell produced anti-microbial peptides [164] and antibodies [165]. It is important to note that the LicA gene that codes for hiChoK is upregulated during the colonization steps of *H. influenzae* [166]. *P*Cho is also important for pathogenesis of *H. influenzae*, because this molecule mediates cell adhesion to the host PAF receptor [162] in a step which leads to invasion the respiratory tract [149]. In addition, *P*Cho decoration of lipoteichoic acid has been shown to reduce the binding of a bacteriocidal antibodies [164].

## 10. Rheumatoid Arthritis and Inflammation

In animal models of rheumatoid arthritis (RA), ChoKIαs are also very successful, suggesting that these drugs have the potential to be used as potent therapeutics in inflammatory diseases [167]. Cell migration and resistance to apoptosis of cultured fibroblast-like synoviocytes (FLS), which are involved in cartilage destruction in RA, were suppressed by **MN58b**, a powerful specific ChoKαI. ChoKα inhibition with **MN58b** significantly decreased FLS migration and proliferation, and abrogated joint inflammation and damage in either pretreatment or proven disease protocols in the K/BxN arthritis mouse model [167].

These findings are in keeping with studies that postulate that synovial inflammation, hyperplasia, and joint destruction are all hallmarks of RA [168]. Phosphoinositide 3-kinase (PI3K)/Akt and Mitogen-activated protein kinase (MAPK) are involved in the control of FLS activity in RA, including matrix metalloproteinases (MMP) expression, synoviocyte growth and survival, and are the subject of therapeutic intervention in RA [169]. As previously mentioned, **MN58b** selectively inhibits ChoKα, which inhibits MAPK and PI3K/Akt signalling [103,104,105], while PI3K inhibition affects ChoK function [170]. Finally, disruption of ChoKα activity affects phosphorylation of Rb, downmodulates cyclin D1, and interferes with PDGF signaling in FLS, as previously demonstrated in other cell systems [62].

Using the *C. Elegans* as a model for Parkinson’s disease (PD), it has been suggested that ChoK may be an important player in PD since inhibition of its expression by siRNA significantly reduces the nuclear localization of Daf-16. ChoK silenced worms showed decreased lipid content, a parameter of immense importance in PD-associated endpoints [113].

Furthermore, ChoKα inhibitors have been shown to be very active in the treatment of several animal models for human diseases related to dysregulation of the inflammasome [171]. Thus, in the LPS-induced septic shock model, ChoKα inhibition dramatically reduced LPS-induced death, an effect associated with reduced levels of circulating IL-1β. Additionally, ChoKα inhibition has therapeutic effects in three models of chronic syndromes associated with cryopyrin (CAPS): Muckle–Wells syndrome (MWS), familial cold autoinflammatory syndrome (FCAS) and neonatal-onset multisystem inflammatory disease (NOMID). These three syndromes are a consequence of mutations in the NLRP3 gene that cause chronic activation of the inflammasome [171]. Therefore, ChoKα inhibition may play an important role in the treatment of a diversity of human diseases related to an altered inflammatory response.

## 11. Future Perspectives: ChoKIs Development for the Treatment of Cancer, Arthritis, Inflammation, Infections and Beyond

ChoKα is overexpressed in a large number of human tumors, and blocking its function causes cancer cells to die while non-tumorigenic cells undergo a reversible cell cycle arrest. Based on a novel mechanism of action, it makes possible combination of ChoK inhibitors with many current therapeutic approaches. Thus, specific targeting of this enzyme is a novel strategy for the treatment of many cancer types. Other diseases for which ChoKα inhibitors have been shown to be effective include malaria, rheumatoid arthritis, inflammation, parasites, and pathogenic bacteria.

One of our ChoKα inhibitors, **RSM932A/TCD717**, has reached the first in human Phase I clinical trial [79]. This first step towards the use of this novel family of drugs in the regular clinical practice has been a milestone that has paved the way for future development of successful drugs. Although further work is needed to finally reach the clinic as a standard of care in any of the several pathological conditions where ChoKIs have been proposed as a therapeutic strategy, the most important issues as the preclinical and the toxicology studies have been already addressed.

There is limited research into ChoKIs of parasites and therefore a limited number of promising ChoK drug leads. Likewise, much of this research has not yet reached the pre-clinical stage, nor therefore Phase I trials. Given this dearth in research, repurposing hChoK inhibitors may be good strategy for discovering novel treatments for parasites. As hChoKIs become cleared for use in humans to treat pathologies such as cancer, rheumatoid arthritis and inflammation, the immense amount of research required to reach that point can be exploited to find new treatments for illnesses such as malaria.

Despite the importance of hiChoK in pathogenesis of *H. influenzae*, the idea that hiChoK could function as a therapeutic target, remains untested. While, hiChoK has not yet been identified as an enzyme critical for cell growth, it is likely that inhibitors for this enzyme could be developed and employed as therapeutic adjuncts designed to assist the immune system in defending against infection.

Meanwhile, the improvements in sChoK inhibition observed with **MN58b** and **RSM932A** fully validated the idea that inhibitors of eukaryotic ChoKs could be repurposed for use against prokaryotes [172,173]. However, sChoK still needs to be fully validated as the drug target responsible for these physiological effects and further metabalonomic and/or mutational studies need to be carried to fully establish this system.

In addition, more refined research needs to be carried out to fully distinguish the effects that these drugs might have on other choline binding proteins such as the autolysin LytA and many others [156]. The autolysin LytA mediates cell autolysis in *S. pneumoniae*, therefore, as an initial step, any putative sChoK inhibitor should also be assayed for its ability to modulate the autolytic process [171,172]. For example, HC-3 is known to modulate autolysis [174], but **MN58b** and **RSM932A** do not. Precision about the actual drug targets is necessary. However, more than one target for putative sChoK inhibitor would actually be highly beneficial. Multiple disruptions in more than one part of the choline metabolism or choline binding pathway may help prevent the development of new resistance to this novel family of inhibitors.

ChoK and other CBPs may be produced by other many bacterial species. Therefore, ChoKIs, like many other drugs [175] may affect species found in the human microbiome. Future studies will need to take these possible effects into account in determine the effects of ChoKIs on human health. The final goal should be to develop a ChoKI therapeutic that is specific to microbial choline kinases, and preferably targeted against specific pathogenic species. Preventing unintended consequences will require a more complete and rationalized understanding of the structural differences between the different ChoKs and CPBs of the various species.

Recently, a functional relationship between the immune checkpoint programmed cell death protein-1 (PD-1) and ChoKα has been established [176]. PD-L1 regulates metabolism through ChoKα, COX-2, and TGF-β, a new twist in the application of combinatorial therapies targeting both the immune checkpoints and modulation of critical enzymes involved in cancer metabolism. Thus, a new door has been opened to further investigate a potential relationship of ChoKα and the immune response.

Finally, since the mechanism of action of ChoKα inhibitors is based on the inhibition of the production of the ILs responsible for these inflammatory processes [166,170] there is another potential application. ChoKαIs reduce macrophage activation and NLRP3 inflammasome attenuating the inflammatory response by modulating the production of IL-6, IL-1β and IL-18. All these processes are carried out by inhibiting ChoKα, the therapeutic target of our inhibitors. COVID-19 patients present in its most acute phases the Severe Acute Respiratory Syndrome (SARS), caused by strong bilateral lung inflammation, responsible for their death. This inflammatory process is associated with a strong increase in cytokine levels mediated by the inflammasome [177] process that coincides with studies carried out with SARS-Cov in which a relationship with the activation of the NLRP3 inflammasome was observed [178]. Based on these observations, it is reasonable to assume that inhibition of the phosphatidylcholine synthesis pathway through inhibition of the ChoKα enzyme could be an effective treatment in COVID-19 patients in the most advanced stages of the disease. This also deserves further investigation.

## Figures and Tables

**Table 1:** Compounds **2**–**12**: structures, biological effects and parameter values.

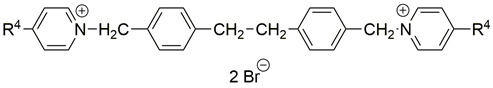						
Comp.	R^4^	(IC_50_)_ex vivo_ (μM) ^a^	(IC_50_)_HT-29_ (μM) ^a^	σ*_R_* ^b^	clog *P* ^c^	π_R4_^d^
**2, MN58B**	–NMe_2_	17.0	2.00	−0.88	−2.83	0.18 ^e^
**3**	–NH_2_	23.0	4.00	−0.80	−4.67	−1.23 ^e^
**4**	–CH_2_OH	100	>100	−0.07	−4.25	−1.03 ^e^
**5**	–CH_3_	100	20.0	−0.16	−1.82	0.56 ^e^
**6**	–COOH	136.7	>1000	0.11	−3.50	−0.32 ^e^
**7**	–C≡N	>1000	200	0.08	−3.15	−0.57 ^e^
**8**	–N(Allyl)_2_	17.0	0.55	−0.80 ^f^	−0.44	1.34 ^e^
**9**	-pyrrolidino	20.0	1.00	−0.85 ^f^	−1.94	0.59 ^e^
**10**	-piperidino	9.60	0.40	−0.89 ^f^	−0.93	0.85 ^e^
**11**	-perhydroazepino	15.0	0.40	−0.86 ^f^	0.09	1.60 ^e^
**12**	–NMePh	6.4	0.34	−0.78 ^f^	0.37	1.67 ^e^

^a^ All values are the mean of two independent determinations performed in duplicate. ^b^ Recombinant human ChoK was used as a target. ^c^ in vitro assay carried out on the HT-29 cell line. ^b^ σ*_R_*: Electronic parameter for resonance effects (ref. [69]). ^c^ Predicted by using the Ghose-Crippen modified atomic contribution system (ATOMIC5 option, ref. [67]) of the PALLAS 2.0 program [68]. ^d^ π_R4_ = clog *P*_R4_ − clog *P*_H_; clog *P* values have been calculated using the CDR option of PALLAS 2.0 program. ^e^ See ref. [70]. ^f^ These values were estimated by ^13^C NMR spectroscopy (see ref. [71]).

**Table 2:** Structures, activity data and parameter used for the generation of QSAR Equation (3).

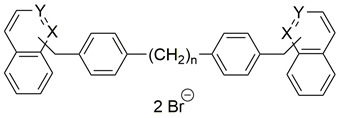						
Comp.	X	Y	*n*	(IC_50_)_ex vivo_ (μM) ^a^	(IC_50_)_HT-29_ (μM) ^a^	Clog *P* ^b^
**13**	=N^+^–	=CH–	0	50	10	−
**14**	=N^+^–	=CH–	1	100	6.02	−0.86
**15**	=N^+^–	=CH–	2	34	4	−0.43
**16**	=N^+^–	=CH–	3	9	2.5	0.08
**17**	=CH–	=N^+^–	0	>100	ND ^c^	−0.85
**18**	=CH–	=N^+^–	1	60	20	−1.00
**19**	=CH–	=N^+^–	2	60	20	−0.57
**20**	=CH–	=N^+^–	3	20	2	−0.06

^a^ All values are the mean of two independent determinations performed in duplicate. ^b^ Recombinant human ChoK was used as a target. ^c^ in vitro assay carried out on the HT-29 cell line. ^b^ Predicted by using the Ghose-Crippen modified atomic contribution system (ATOMIC5 option, ref. [67]) of the PALLAS 2.0 program [68]. ^c^ ND: Not determined.

**Table 3:** Compounds **21** and **22**: structures, biological effects and parameter values.

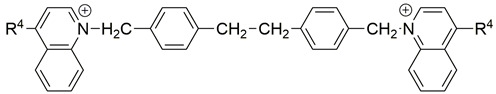						
Comp.	R^4^	(IC_50_)_ex vivo_ (μM) ^a^	(IC_50_)_HT-29_ (μM) ^a^	σ*_R_* ^b^	clog *P* ^c^	π_R4_^d^
**21**	–NH_2_	10.0	2.00	−0.80	−2.13	−1.23
**22**	–NHCOBu*^t^*	10.5	4.74	−0.35 ^e^	1.40	2.18

^a^ All values are the mean of two independent determinations performed in duplicate. ^b^ Recombinant human ChoK from yeast was used as a target. ^c^ in vitro assay carried out on the HT-29 cell line. ^b^ σ*_R_*: Electronic parameter for resonance effects (ref. [69]). ^c^ Predicted by using the Ghose-Crippen modified atomic contribution system (ATOMIC5 option, ref. [67]) of the PALLAS 2.0 program [68]. ^d^ π_R4_ = clog *P*_R4_ − clog *P*_H_; clog *P* values have been calculated using the CDR option of the PALLAS 2.0 program. ^e^ We have used the acetamido value instead of the pivaloylamino group σ*_R_* value because the latter value was unavailable (ref. [69]).

**Table 4:** IC_50_ ChoK and HT-29 values for the bisquinolinium derivatives belonging to series **A**.

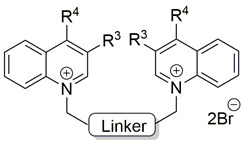					
Comp.	Linker	R^3^	R^4^	(IC_50_)_ex vivo_ (μM) ^a,b^	(IC_50_)_HT-29_ (μM) ^a,c^
**23**	Biphenyl-3,3′-diyl	H	Amino	1.20	1.90
**24**		Me	Amino	11.9	4.40
**25**		H	Dimethylamino	4.40	1.60
**26**		H	Perhydroazepino	0.50	0.50
**27**		H	Anilino	1.30	1.60
**28**		H	*N*-Methylanilino	0.40	0.80
**29**		H	4-Chloro-*N*-methylanilino	2.10	1.50
**30**	Biphenyl-4,4′-diyl	H	Amino	81.1	2.20
**31**		Me	Amino	>200	3.30
**32**		H	Dimethylamino	39.7	1.70
**33**		H	Perhydroazepino	2.20	0.50
**34**		H	Anilino	17.8	0.70
**35**		H	*N*-Methylanilino	3.00	0.60
**36**		H	4-Chloro-*N*-methylanilino	2.00	1.20
**37**	Bibenzyl-4,4′-diyl	H	Amino	80.0	2.00
**38**		H	Dimethylamino	10.2	0.50
**39**		H	Perhydroazepino	0.60	0.30
**40**		H	Anilino	2.30	0.30
**41**		H	*N*-Methylanilino	1.40	0.70
**42**		H	4-Chloro-*N*-methylanilino	4.80	0.70

^a^ All values are the mean of two independent determinations performed in duplicate. ^b^ Recombinant human ChoK was used as a target. ^c^ in vitro assay carried out on the HT-29 cell line.

**Table 5:** IC_50_ ChoK and HT-29 values for the bisquinolinium derivatives belonging to series **B**.

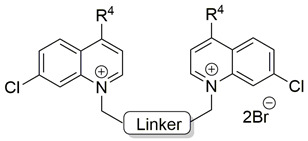				
Comp.	Linker	R^4^	(IC_50_)_ex vivo_ (μM) ^a,b^	(IC_50_)_HT-29_ (μM) ^a,c^
**43**	Biphenyl-3,3′-diyl	Amino	20.6	1.90
**44**		Dimethylamino	9.60	0.70
**45**		Pyrrolidino	1.20	0.40
**46**		*N*-Methylanilino	3.10	1.00
**47**		4-Chloro-*N*-methylanilino	5.70	1.90
**48**	Biphenyl-4,4′-diyl	Amino	63.3	3.20
**49**		Dimethylamino	20.6	0.80
**50**		Pyrrolidino	19.8	2.40
**51**		*N*-Methylanilino	11.4	0.50
**52, RSM-932A**		4-Chloro-*N*-methylanilino	11.4	1.20
**53**	Bibenzyl-4,4′-diyl	Amino	80.0	2.00
**54**		Dimethylamino	9.00	0.27
**55**		Pyrrolidino	1.00	0.20
**56**		*N*-Methylanilino	3.50	0.50
**57**		4-Chloro-*N*-methylanilino	5.70	0.80

^a^ All values are the mean of two independent determinations performed in duplicate. ^b^ Recombinant human ChoK was used as a target. ^c^ in vitro assay carried out on the HT-29 cell line.

**Table 6:** IC_50_ ChoK and HT-29 values for the bisquinolinium derivatives belonging to series **C**.

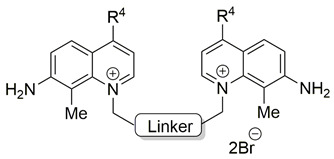				
Comp.	Linker	R^4^	(IC_50_)_ex vivo_ (μM) ^a,b^	(IC_50_)_HT-29_ (μM) ^a,c^
**58**	Biphenyl-3,3′-diyl	*N*-Methylanilino	56.8	3.80
**59**		4-Chloro-*N*-methylanilino	147	33.0
**60**	Biphenyl-4,4′-diyl	*N*-Methylanilino	96.1	25.2
**61**		4-Chloro-*N*-methylanilino	46.1	19.4
**62**	Bibenzyl-4,4′-diyl	*N*-Methylanilino	133	7.00
**63**		4-Chloro-*N*-methylanilino	57.5	19.5

^a^ All values are the mean of two independent determinations performed in duplicate. ^b^ Recombinant human ChoK was used as a target. ^c^ in vitro assay carried out on the HT-29 cell line.

## Data Availability

No new data were created or analyzed in this study. Data sharing is not applicable to this article.

## Abbreviations

ChoKα, ChoKβ	Choline Kinase α or β
PC	phosphatidylcholine
*P*Cho	phosphorylcholine
ChoKI	ChoK inhibitor
ChoKαI	ChoKα-specific inhibitor
siRNA	small interfering RNA
5-FU	5-Fluorouracil
PI3K	phosphatidylinositol 3 kinase
MAPK	mitogen activated protein kinase
EGFR	epidermal growth factor receptor
NMR	nuclear magnetic resonance
MRS	magnetic resonance spectroscopy
PET	positron emission tomography
HR-MAS MRS	high-resolution magic angle spinning magnetic resonance spectroscopy
FLS	fibroblast-like synoviocytes
RA	rheumatoid arthritis
